# *In Vitro* and *in Vivo* Effects of *Laurus nobilis* L. Leaf Extracts

**DOI:** 10.3390/molecules15053378

**Published:** 2010-05-07

**Authors:** Biljana Kaurinovic, Mira Popovic, Sanja Vlaisavljevic

**Affiliations:** Department of Chemistry, Faculty of Science, University of Novi Sad, Trg Dositeja Obradovica 3, 21000 Novi Sad, Serbia

**Keywords:** *Laurus nobilis* L., extracts, free radicals, *in vitro* experiments, biochemical parameters

## Abstract

The *in vitro* and *in vivo* antioxidant activities of different extracts of laurel leaves were studied. Free radical scavenging capacity (RSC) was evaluated measuring the scavenging activity on the DPPH, NO, O_2_^•^^−^ and OH radicals. The effects on lipid peroxidation (LP) were also evaluated. Experimental results indicate that ethyl acetate extract of leaves has exhibited the largest RSC capacity in neutralization of DPPH, NO, O_2_^•^^−^ and OH radicals. The same result was obtained in investigation of extracts impact on LP. The *in vivo* effects were evaluated on some antioxidant systems (activities of GSHPx, LPx, Px, CAT and XOD, and GSH content) in the mice liver and blood-hemolysate after treatment with the examined laurel extracts, or in combination with carbon tetrachloride (CCl_4_). On the basis of the results obtained it can be concluded that the examined extracts exhibited a certain protective effect, which is more pronounced on the liver than on blood-hemolysate parameters. The results obtained indicate toxicity of CCl_4_, probably due to the radicals involved in its metabolism. Combined treatments with CCl_4_ and the examined extracts showed both positive and negative synergism. Based on the experimental results, the strongest protective effect was shown by the EtOAc extract.

## 1. Introduction

The Lauraceae family comprises over 2,500 species which occur within the subtropics and tropics of Eastern Asia, South and North America. Most species possess aromatic roots, stems and fruits. One of the most well-known and most frequently used plants from this family is *Laurus nobilis* L., also called bay laurel. *L. nobilis* is a species held in high esteem since ancient times. It was dedicated to Apollo, the ancient Greek god of light, and a symbol of peace and victory used to make wreaths for emperors, generals, and poets [1].

The chemical composition of bay leaves has been extensively studied. From the essential oil, also called laurel leaf oil, various volatile components with antimicrobial activities against bacteria, yeasts, and some molds were identified [2,3,4]. 1,8-Cineole was the major component in all cases, with percentages ranging up to ca. 50% [5]. The presence of linalool, *R*-terpinyl acetate, and several monoterpene hydrocarbons such as α-pinene and sabinene was also determined. Benzene compounds (eugenol, methyl eugenol, and elemicin), present in percentages ranging between 1% and 12%, are responsible for the spicy aroma of bay leaves and are extremely important factors determining the sensory quality of bay leaves [6]. The berries (fruit) contain both fixed and volatile oils, the former, known as Oil of Bays, include laurostearine, the ether of lauric acid.

In previous phytochemical investigations on *L. nobilis* leaves and fruits, different groups of chemicals were isolated: flavones (apigenin and luteolin) [7], flavonols (kaempferol, myricetin, and quercitin) [8], sesquiterpene lactones [9], alkaloids [10], glycosylated flavonoids [11], and mono-terpene and germacrane alcohols [12]. 

Roots and leaves are a source of sesquiterpene lactones, and two distinct chemical types were found containing laurenobiolide and costunolide as major compounds, respectively [13,14]. Sesquiterpene lactones identified in bay leaf were found to have different pharmacological properties including inhibitory effects on NO production (anti-inflammatory) [15], inhibitory effects on alcohol absorption [16], and enhancement of liver glutathione *S*-transferase (GST) activity [17]. 

As a medicinal plant, Bay leaves and fruits have been employed against rheumatism, skin rashes, and earaches. In addition, it has been used as a stomachic, astringent, carminative, diaphoretic, stimulant, emetic, emmenagogue, abortifacient, and insect repellent. The essential oil is used by the cosmetic industry in creams, perfumes, and soaps. 

The aim of this work was to study the antioxidant activity of different extracts of laurel leaves *in vitro*, to assess their potential capacity as scavengers of free radicals (DPPH, NO, O_2_^•^^− ^and OH radicals) and as inhibitors of lipid peroxidation. Extracts of laurel leaves were used to study *in vivo* effects on some antioxidant systems in the mice liver and blood in combination with and without carbon tetrachloride (CCl_4_) as an inducer of oxidative stress.

## 2. Results and Discussion

Results of determination of total flavonoids in laurel extracts of leaves are given in Table 1.

**Table 1:** Content of total flavonoids (mg/g) in extracts of leaves of *L. nobilis*.

Extract	Et_2_O	CHCl_3_	EtOAc	n-BuOH	H_2_O
**Leaf **	0.76	1.02	1.56	1.07	0.68

The most abundant in flavonoids proved to be the ethyl acetate extract, while water extract contains the least flavonoids. Amount of flavonoids in extracts plays a significant role in their antioxidative capacity. Differences in flavonoid content between extracts and between plant organs can be explained by different number of secretory structures in various plant tissues [18].

Antiradical activity was observed in the study of laurel leaves extracts in different solvents on the content of DPPH, O_2_^•^^−^ and NO radicals (Table 2.), whereby the EtOAc extract exhibited the strongest inhibitory effect, as the IC_50_ value was achieved with the lowest concentration. 

**Table 2:** IC_50_ values (μg/cm^3^) of the neutralization of DPPH, O_2_^•^^−^ and NO radicals with laurel extracts.

		IC_50_ (μg/cm^3^)			
Extract	Et_2_O	CHCl_3_	EtOAc	n-BuOH	H_2_O
**DPPH radical**	127.38	139.42	83.24	181.35	161.83
**O_2_^•^^−^ radical**	327.60	429.43	163.57	288.64	486.32
**NO radical**	168.77	322.84	158.63	386.80	618.42

The obtained results could point to strong quenching activities of flavonoids present in the leaves of laurel against DPPH radicals [19]. It can be supposed that such antiradical activity is also caused, besides flavonoids, by terpenoids, since nonpolar solvents also exhibited high antiradical potential. When investigating neutralization of O_2_^•^^−^ and NO radicals, ethylacetate extract has also exhibited the greatest ability of their scavenging. These results can be attributed to the presence of sesquiterpenic lactones isolated from the plant that possess certain biological and pharmacological activity [20,21]. Matsuda *et al*. [15] have also established that the methanolic extract from the leaves of *L. nobilis* was found to inhibit nitric oxide (NO) production in lipopolysaccharide (LPS)-activated mouse peritoneal macrophages. It was concluded that seven sesquiterpene lactones (costunolide, dehydrocostus lactone, eremanthine, zaluzanin C, magnolialide, santamarine and spirafolide) potently inhibited LPS-induced NO production. 

Inhibition of NO radicals with laurel extracts is very significant, having in mind the ability to neutralize the superoxide anion radicals as well. The common reaction between superoxide anion radical and nitrogen-oxide radical yields a very reactive peroxynitrile anion (ONOO^−^) which is very active in reaction of nitrification of phenols - e.g. nitrification of thyrozine causes enzyme disfunctions, and increased amounts of 3-nitrothyrozine were found in various patological states, such as inflammation, Parkinson's disease, Alzheimer's disease, and atherosclerosis [22]. If formation of nitro-derivatives of thyrozine is prevented, the occurrence of these diseases due to oxidative stress is reduced. Ethyl acetate extract of laurel leaves is especially suited in this process since it neutralizes both superoxide anion radical and NO radical. Obtained results can be related to the experiments in which the total amount of flavonoids was determined (Table 1.), which show that ethyl acetate extract of laurel leaf contains the largest amounts of total flavonoids. 

The activity of the laurel extracts (1%, 5% and 10%) on the production of hydroxyl radicals is shown in Table 3. as mean with standard deviation. Laurel extracts have exhibited different behavior related to production of OH radicals. Ether, chloroform and ethyl acetate extracts expressed inhibition of OH radical at all investigated concentrations. A stronger inhibition of OH radical production was expressed by the ethyl acetate extracts, especially the 10% solution (26.5 ± 1.1 nmol/cm^3^) in comparison with the control (51.4 ± 1.2 nmol/cm^3^). *n*-Butanol and water extract have prooxidative effects, but not statistically significant. The antioxidant activities of all extracts of laurel leaves were dose dependent.

**Table 3:** Effect of extracts of laurel leaves on production of hydroxyl radicals.

Extract	Control	Et_2_O	CHCl_3_	EtOAc	n-BuOH	H_2_O
**1%**	51.4 ± 1.2	47.7 ± 1.1 (7.2)	44.5 ± 1.2 (13.4)	42.5 ± 0.9 (17.3)	51.8 ± 1.2	52.7 ± 1.0
**5%**	51.4 ± 1.2	41.7 ± 1.2 (18.9)	38.3 ± 1.1 (25.5)	34.7 ± 1.1 (32.5)	53.2 ± 1.0	53.8 ± 1.1
**10%**	51.4 ± 1.2	35.2 ± 1.4 (31.5)	33.5 ± 1.3 (34.8)	26.5 ± 1.1 (48.4)	53.6 ± 1.2	54.7 ± 1.4

Results are expressed in nmol/cm^3 ^of liposomes. Values in the parentheses represent % of inhibition of hydroxyl radical production.

Since lipid peroxidation causes oxidative damage to cell membranes and all other systems that contain lipids [23], in investigation of total antioxidative activity of extracts it is necessary to investigate their effects on lipid peroxidation (Table 4.). The first three extracts of laurel leaves (Et_2_O, CHCl_3_ and EtOAc) reduced the intensity of lipid peroxidation. The largest inhibitory activity, again, was exhibited by ethyl acetate extract. High inhibitory effect of this extract and its components towards Fe^2+^-dependent LP of liposomes can be related to the presence of flavonoids in the extracts. It was established that flavonoids act as mighty scavangers of free radicals [24]. It is also known that antiradical potential of flavonoids are the most pronounced towards OH, peroxy- and alkoxy radicals, which are formed in the process of lipid peroxidation [25]. Different flavonoids inhibit LP *in vitro* and the most pronounced effect is exhibited by quercetin [26]. Our previous investigation has also established similar results, since the ethyl acetate extract of marigold has also statistically significantly decreased production of OH radicals [27]. Since quercetin and its glycosides are the most abundant flavonoids in both marigold and laurel [8], efficiency of above mentioned ethyl acetate extracts can be explained in two ways: components of the ethyl acetate extract may inhibit production of OH radicals in the Fenton’s reaction system Fe^2+^-H_2_O_2_, by forming complexes with Fe^2+^ ions. It is well known that flavonoids containing free 5-OH groups form complexes with bi- and trivalent metal ions [28]. Also, components of the extract can act as scavengers of OH radicals, thus forming new radicals, wich can be stabilized through resonance [29].

**Table 4:** Effect of extracts of laurel leaves on intensity of lipid peroxidation.

Extract	Control	Et_2_O	CHCl_3_	EtOAc	n-BuOH	H_2_O
**1%**	144.9 ± 3.6	138.1 ± 1.9 (4.7)	137.8 ± 1.4 (4.9)	130.4 ± 2.6 (10.0)	163.8 ± 3.1	165.3 ± 2.8
**5%**	144.9 ± 3.6	130.3 ± 2.2 (10.1)	128.8 ± 1.1 (11.1)	116.9 ± 1.9 (19.3)	166.3 ± 2.3	172.3 ± 2.4
**10%**	144.9 ± 3.6	125.1 ± 3.1 (13.7)	123.8 ± 2.4 (14.6)	105.4 ± 1.6 (27.3)	170.8 ± 2.4	179.3 ± 2.1

Results are expressed in nmol/cm^3 ^liposomes. Values in the parentheses representing % of inhibition of lipid peroxidation.

Two other extracts (*n*-BuOH and H_2_O extracts) have prooxidative effects. Investigations of the potential free radical scavenging activity of the examined extracts on different radicals showed that not the same substances are responsible for the antioxidant effects. This difference in antioxidant activity was recently also shown for some essential oils and isolated terpenoids [30,31]. 

In accordance with the results of the antioxidant effects of the extracts of laurel leaves obtained in *in vitro* assays, examinations of the *in vivo* activity of Et_2_O, CHCl_3_, EtOAc, *n*-BuOH and H_2_O extracts of laurel leaves (solutions made in 50% of ethanol) were conducted. The experimental animals were given 1 cm^3^/kg of 2% of Et_2_O, CHCl_3_, EtOAc, *n*-BuOH or H_2_O extract (i.p.) of laurel leaves for 5 days. After 5 days, the animals were killed. In the liver homogenate and blood hemolysate of killed animals the following biochemical parameters were determined: LPx intensity, content of GSH and activities of GSHPx, Px, XOD and CAT (Table 5 and Table 7). In Table 6 and Table 8 the results of the same parameters obtained after pretreatment of mice with the examined laurel extracts, followed by a single dose of carbon tetrachloride (CCl_4_) as a well-known radical generator are presented.

As can be seen from Table 5 the Et_2_O extract and H_2_O extracts decreased the GSH content compared with control. The exceptionally high GSH values in the treatments with the EtOAc and especially n-BuOH extracts is probably due to the involvement of –SH groups from some other compounds apart from glutathione. Namely, the GSH content can not be significantly higher than for control, as about 95% of GSH in cytosol is found as free. The GSHPx activity decreased in the treatment with Et_2_O and CHCl_3_ extract, H_2_O extract caused its increase, while the other two extracts caused no essential changes of this parameter. With the exception of the Et_2_O extract, all other extracts decreased the extent of LPx, which indicates that they exhibit an antioxidant effect, *i.e.* they contain antioxidative compounds. The Px activity increased in the CHCl_3_ and H_2_O treatments and decreased in the treatment with the EtOAc extract. The CAT increased in the treatments with EtOAc and H_2_O extracts, the other extracts caused no essential changes of CAT with respect to control. An increased XOD value was observed only in the treatments with the Et_2_O extract.

**Table 5:** Effect of extracts of laurel leaves on the biochemical parameters in the liver homogenate.

Parameter	Control	Et_2_O extract	CHCl_3_ extract	EtOAc extract	*n*-BuOH extract	H_2_O extract
GSH	2.13 ± 0.16	2.02 ± 0.18	2.26 ± 015	2.76 ± 0.26^a^	3.04 ± 0.23^a^	1.87 ± 0.16
GSHPx	2.21 ± 0.17	1.74 ± 0.13^a^	1.65 ± 0.18^a^	2.13 ± 0.17	2.08 ± 0.24	2.72 ± 0.18^a^
LPx	2.56 ± 0.22	3.18 ± 0.21^a^	2.12 ± 0.23	1.87 ± 0.12^a^	2.06 ± 0.21^a^	2.03 ± 0.23^a^
Px	5.89 ± 0.24	5.81 ± 0.23	6.48 ± 0.25^a^	4.93 ± 0.23^a^	5.58 ± 0.23	6.52 ± 0.25^a^
CAT	3.36 ± 0.26	3.48 ± 0.21	3.28± 0.19	3.87 ± 0.17^a^	3.11 ± 0.18	3.75 ± 0.19^a^
XOD	4.20 ± 0.24	4.89 ± 0.25^a^	3.78 ± 0.16^a^	2.96 ± 0.18^a^	4.21 ± 0.26	3.47 ± 0.25^a^

t-test ^a^ p ≤ 0.05 n = 6; x ± SD. Content of GSH is expressed in nmol GSH/mg of protein. Activities of GSHPx, Px, CAT and XOD are expressed in nmol/mg of protein x min^-1^. Intensity of LPx is expressed in nmol malondialdehyde/mg of protein.

As could be expected, treatment with CCl_4_ decreased the liver GSH content (Table 6), as this agent acts as a pro-oxidant, and GSH decrease is one of the ways of the organism′s antioxidative defense [32]. Obviously, by decreasing GSH content CCl_4_ impaired antioxidant status in hepatocytes. In previous studies, Mimica-Dukic *et al*. [33] found that flavonoids and phenolic acids from leaves of *Mentha longifolia* diminished the CCl_4_-mediated depletion of GSH. In the present study none of the examined extracts inhibited GSH depletion. Namely, with the exception of the Et_2_O one, all the extracts increased the GSH content compared to the control and yielded the same results as in Table 5, which indicates increased contents of secondary biomolecules exhibiting antioxidant effect.

**Table 6:** Effect of laurel leaves extracts and CCl_4 _on the liver homogenate biochemical parameters.

Parameter	Control	Et_2_O extract	CHCl_3_ extract	EtOAc extract	*n* -BuOH extract	H_2_O extract
		+ CCl_4_	+ CCl_4_	+ CCl_4_	+ CCl_4_	+ CCl_4_
GSH	1.94 ± 0.14	1.16 ± 0.11^a^	2.80 ± 0.18^a^	2.04 ± 0.27	2.08 ± 0.15	1.94 ± 0.22
GSHPx	3.25 ± 0.15	3.88 ± 0.17^a^	3.72 ± 0.18^a^	2.47 ± 0.22^a^	3.06 ± 0.24	3.17 ± 0.18
LPx	2.85 ± 0.22	3.76 ± 0.11^a^	3.79 ± 0.21^a^	1.46 ± 0.12^a^	2.91 ± 0.21	1.41 ± 0.17^a^
Px	5.82 ± 0.24	6.78 ± 0.23^a^	7.26 ± 0.25^a^	5.91 ± 0.17	5.87 ± 0.20	7.31 ± 0.25^a^
CAT	3.24 ± 0.26	3.88 ± 0.21^a^	3.66 ± 0.19	3.74 ± 0.17^a^	3.82 ± 0.18^a^	3.88 ± 0.19^a^
XOD	2.62 ± 0.14	2.96 ± 0.17	3.03 ± 0.21^a^	2.19 ± 0.18^a^	2.91 ± 0.26	2.27 ± 0.17^a^

t-test ^a^ p ≤ 0.05 n = 6; x ± SD. Content of GSH is expressed in nmol GSH/mg of protein. Activities of GSHPx, Px, CAT and XOD are expressed in nmol/mg of protein x min^-1^. Intensity of LPx is expressed in nmol malondialdehyde/mg of protein.

It has been proposed that one of the principal causes of CCl_4_-induced liver injury is lipid peroxidation induced by free radicals derivatives of CCl_4_ [34]. Increased LPx values were observed with the CCl_4_-treated animals compared to control. The EtOAc and H_2_O extracts in combination with CCl_4_ showed protective effect whereas the others showed a synergistic pro-oxidative effect.

Treatments with Et_2_O and CHCl_3_ extracts in combination with CCl_4_ resulted in increased GSHPx values, while EtOAc extract produced its decrease. At the same time, the Px activity did not essentially change compared with the control (5.82 ± 0.24 *vs*. 5.89 ± 0.24). Treatments with all extracts in combination with CCl_4_ yielded an increased CAT value compared with the control. At the same time, treatments with Et_2_O, CHCl_3_ and n-BuOH extracts combined with CCl_4_ resulted in increased XOD values, while EtOAc and H_2_O caused their decrease.

Decreased LPx values were observed in treatments with CHCl_3_, EtOAc and H_2_O extracts, the other extracts caused no essential changes (Table 7). All laurel extracts produced increases of Px and CAT, while XOD activity decreased in the treatments with Et_­2_O and EtOAc extracts and increased in the other treatments.

**Table 7:** Effect of extracts of laurel leaves on the biochemical parameters in blood hemolysate.

Parameter	Control	Et_2_O extract	CHCl_3_ extract	EtOAc extract	*n*-BuOH extract	H_2_O extract
GSH	7.26 ± 0.27	6.83 ± 0.21	7.18 ± 0.17	7.21± 0.24	7.56 ± 0.31	7.41 ± 0.22
GSHPx	6.11 ± 0.23	4.13 ± 0.17^a^	5.82 ± 0.18	3.84 ± 0.17^a^	5.95 ± 0.25	6.71 ± 0.27^a^
LPx	7.61 ± 0.22	7.82 ± 0.21	6.11 ± 0.31^a^	4.83 ± 0.22^a^	7.35 ± 0.23	5.14 ± 0.19^a^
Px	4.97 ± 0.14	5.48 ± 0.17^a^	5.31 ± 0.15^a^	5.57 ± 0.17^a^	5.33 ± 0.30	5.59 ± 0.25^a^
CAT	4.18 ± 0.26	5.51 ± 0.21^a^	4.75 ± 0.19^a^	4.26 ± 0.24	4.58 ± 0.18	4.79 ± 0.17^a^
XOD	4.78 ± 0.14	3.89 ± 0.17^a^	5.96 ± 0.26^a^	3.77 ± 0.18^a^	5.18 ± 0.26^a^	5.26 ± 0.25^a^

t-test ^a^ p ≤ 0.05 n = 6; x ± SD. Content of GSH is expressed in μmol GSH/dm^3^ erythrocytes Activities of GSHPx, Px, CAT and XOD are expressed in nmol/dm^3^ erythrocytes x min^-1^. Intensity of LPx is expressed in nmol malondialdehyde/dm^3^ erythrocytes.

A comparasion of GSH values in Table 7 and Table 8 shows that the GSH value is significantly lower in the animals treated with CCl_4_ (7.26 ± 0.27 *vs.* 5.06 ± 0.27). This indicates that GSH acts as one of the essential antioxidant systems. On the other hand, the extracts showed no protective effect; moreover, all of them produced a further decrease of the GSH value. No statistically significant effect on GSHPx activity was observed in the combined treatment with laurel extracts and CCl_4_, only the EtOAc extract decreased this activity (*p* < 0.001).

**Table 8:** Effect of extracts of laurel leaves and CCl_4_ on the biochemical parameters in blood hemolysate.

Parameter	Control	Et_2_O extract	CHCl_3_ extract	EtOAc extract	*n* -BuOH extract	H_2_O extract
		+ CCl_4_	+ CCl_4_	+ CCl_4_	+ CCl_4_	+ CCl_4_
GSH	5.06 ± 0.27	4.28 ± 0.21^a^	4.86 ± 0.16	4.80 ± 0.17	4.72 ± 0.31	4.11 ± 0.22^a^
GSHPx	8.19 ± 0.23	8.26 ± 0.17	8.14 ± 0.19	6.92 ± 0.27^a^	8.12 ± 0.24	8.34 ± 0.28
LPx	9.42 ± 0.22	8.06 ± 0.21^a^	7.97 ± 0.24^a^	6.17 ± 0.22^a^	6.28 ± 0.25^a^	5.96 ± 0.31^a^
Px	4.35 ± 0.14	2.79 ± 0.23^a^	3.19 ± 0.15^a^	2.79 ± 0.26^a^	2.96 ± 0.30^a^	1.89 ± 0.27^a^
CAT	4.24 ± 0.16	2.61 ± 0.21^a^	3.28 ± 0.19^a^	2.91 ± 0.17^a^	3.14 ± 0.18^a^	2.26 ± 0.23^a^
XOD	3.61 ± 0.14	2.08 ± 0.19^a^	3.56 ± 0.16	3.33 ± 0.18	3.79 ± 0.26	3.72 ± 0.25

t-test ^a^ p ≤ 0.05 n = 6; x ± SD. Content of GSH is expressed in μmol GSH/dm^3^ erythrocytes Activities of GSHPx, Px, CAT and XOD are expressed in nmol/dm^3^ erythrocytes x min^-1^. Intensity of LPx is expressed in nmol malondialdehyde/dm^3^ erythrocytes.

The LPx value showed a statistically significant increase with CCl_4_-treated animals compared with the untreated ones, whereas the presence of laurel extract yielded a decrease of LPx. These results suggest that the extracts had a protective effect. According to the literature data [35], the reduction of the serum LPx might be the result of antioxidant activity of several classes of plant phenolic constituents, such as cinnamic acids (ferulic, caffeic, and chlorogenic), flavonoids and biflavonoids, 1,3,6,7-tetrahydroxyxanthones, and acylphoroglucinols such as hyperforin and adhyperforin. Cock and Samman [28] showed that quercetin and rutin and their glycosides show a strong inhibitory effect in respect of LPx. Very indicative are the results obtained for the Px and CAT, which are significantly decreased in combination of all extracts with CCl_4_. A statistically significant decrease of XOD activity was observed only in the case of treatment with the Et_2_O and EtOAc extracts, the other extracts exhibited no influence on it. 

## 3. Experimental

### 3.1. General

Leaves of cultivated *Laurus nobilis* L., Lauraceae, were collected in June 2008 in the city of Ulcinj, Montenegro. The voucher specimen of the collected leaves (*Laurus nobilis* L. 1753, No 2-1811, Montenegro, Ulcinj (UTM 34T CM 4 54), 05.06.2008., det.: Dr Goran Anačkov) was confirmed and deposited at the Herbarium of the Department of Biology and Ecology (BUNS Herbarium), Faculty of Natural Sciences, University of Novi Sad.

The plant leaves were dried in air and ground in a mixer. Finely powdered material (200 g) was macerated three times in 70% methanol (MeOH) with 4dm^3^ during a 24-h period. The macerates were collected, filtered, and evaporated to dryness under vacuum. The residues were dissolved in water and successively extracted with four solvents of increasing polarity: ether (Et_2_O), chloroform (CHCl_3_), ethyl acetate (EtOAc), and *n*-butanol (*n*-BuOH). The extraction was carried out until a colorless extract was obtained. The residue was the aqueous extract. All of five extracts (Et_2_O, CHCl_3_, EtOAc, *n*-BuOH, and H_2_O) were evaporated to dryness and then dissolved in 50% ethanol to make 10% (w=v) solutions. Both, these and the diluted solutions, were further used for examination. 

RSC was evaluated measuring the scavenging activity of the examined extracts on the 2,2-diphenyl-1-picrylhydrazyl (DPPH), superoxide anion (O_2_^•^^−^), nitrite-oxide (NO) and OH radicals using a Beckman DU 65 spectrophotometer. Also, intensity of LPx in liposomes and biochemical parameters were evaluated using the same unit.

### 3.2. In vitro experiments

Determination of total flavonoids was conducted by colorimetric method based on the property of flavonoids and flavone glycosides to build metal complexes with aluminum ions [36]. Absorbance of analyzed solutions is measured at λ = 430 nm.

The DPPH assay was performed as described before [37], following the transformation of the DPPH radical to its reduced, neutral form (DPPH-H). The samples of all extracts of laurel leaves were investigated in concentrations of 50–500 μg/cm^3^. The disappearance of DPPH was measured spectrophotometrically at 515 nm. 

Superoxide anion radicals were generated in the system xantine/xantine-oxidase, and the quantity of O_2_^•^^−^ was determined by nitrite method [38] with modifications. This system was used also in determination of the level of inhibition of xantine-oxidase. The samples of all extracts of laurel leaves were investigated in concentrations of 50–500 μg/cm^3^. The intensity of color was measured spectrophotometrically (λ = 550 nm).

Production of NO radicals was determined spectrophotometrically. NO radical generated from sodium-nitropruside (SNP) reacts with oxygen in water solution at a physiological pH to give nitrite ions. Concentration of nitrite anions was determined using Griess reagent [39,40]. At room temperature nitrite ions react with Griess reagent and form purple complex. The samples of all extracts of laurel leaves were investigated in concentrations of 50–1,000 μg/cm^3^. The intensity of color, which is the function of the nitrite concentrations, was measured spectrophotometrically (λ = 546 nm). The absorbance of the resulting solutions and the blank (with the same chemicals, except for the sample) were recorded. For each sample, five replicates were recorded. 

The IC_50_ value, which represents the concentrations of the examined extracts that caused 50% of inhibition, was determined by linear regression analysis from the obtained RSC values.

The effect of laurel leaves extracts on the production of OH radicals was determined by monitoring the chemical degradation of deoxyribose [41]. The reaction was initiated by hydroxyl radicals obtained in Fenton’s reaction [42], which yields products that react with thiobarbituric acid (TBA test). The obtained products, among which malondialdehyde (MDA) is the most important, are determined by a spectrophotometric method at 532 nm. The absorbance of the resulting solutions and the blank (with same chemicals, except sample) was recorded. For the experiment, three concentrations of extracts of laurel leaves were prepared (10%, 5% and 1% solution). Five replicates were performed for each sample. The absorbance at the end of the experiment was used to calculate the inhibition rate of deoxyribose degradation by the examined extracts.

The extent of LP was determined by measuring the color of the adduct produced in the reaction between 2-thiobarbituric acid (TBA) and malondialdehyde (MDA), as an oxidation product in the peroxidation of membrane lipids, by the TBA assay [34,43,44]. The commercial preparation of liposomes ‘PROLIPOS’ (Lucas-Meyer) pH 5–7 was used as a model system of biological membranes. The liposomes, 225–250 nm in diameter, were obtained by dissolving the commercial preparation in demineralized water (1:10), in an ultrasonic bath. For the experiment, three concentrations of extracts of laurel leaves were prepared (10%, 5% and 1% solution). Five replicates were performed for each sample.

### 3.3. In vivo *antioxidant activity*

This investigation was conducted on sexually mature White laboratory mice (both sexes), type BALB/C, with an average body weight of 20–28 grams. Animal care and all experimental procedures were conducted in accordance with the *Guide for the Care and Use of Laboratory Animal Resources*, edited by Commission of Life Sciences, National Research Council, Male and female Hanover National Medical Institute (Hann NMRI). Mices were bred in the vivarium at the Department of Pharmacology, Toxicology and Clinical Pharmacology, Medical Faculty, University of Novi Sad, Serbia. Animals were kept in standard plexiglass cages (room temperature 21 ± 1 °C; humidity 55 ± 1.5%, with 12 h light period). They were fed standard laboratory mice feed, produced by the Veterinary Institute in Zemun. Animals were given free access to food and fluid (water). Animals were randomly assigned into six groups, consisting of 10 animals each. The animals of control group were given intraperitoneally (i.p.) physiological solution, the animals of five experimental groups received at the same time (i.p., 1.0 cm^3^/kg) one of five laurel leaf extracts. After 7 days five animals from each group were sacrificed to determine the biochemical liver and blood parameters. The rest six animals of each group were treated (i.p.) with CCl_4_ in olive oil (1:1, 2.0 cm^3^/kg) and sacrificed 24 h later, to determine the same biochemical parameters.

We have measured examined biochemical parameters in blood hemolysate and liver homogenate. Liver was homogenized in a Potter homogenizer with TRIS-HCl/sucrose in a ratio of 1:3 at 4 °C. Obtained homogenate was filtered. The following biochemical parameters were analyzed in blood hemolysate and liver homogenate: extent of lipid peroxidation (LPx) was determined after Buege and Aust [44], peroxidase (Px) activity was measured after Simon *et al*. [45], catalase activity (CAT) after Beers and Sizer [46]. Glutathione peroxidase (GSH-Px) activity was evaluated as described by Chin *et al*. [47], xanthine oxidase (XOD) after Bergmayer [48], content of reduced glutathione (GSH) after Kapetanović and Mieyal [49]. The total protein content in liver was determined after Gornall *et al*. [50].

### 3.4. Chemicals

Thiobarbituric acid (TBA), xanthine, xanthine-oxidase, ethylenediaminetetraacetic acid (EDTA), 2,2-diphenyl-1-picrylhydrazyl (DPPH) were obtained from Sigma Chemicals. 2-deoxy-D-ribose was purchased from Aldrich. DTNB and reduced glutathione was obtained from Merck (Darmstadt, Germany). The commercial preparation of liposomes “PRO-LIPO S” was purchased from Lucas-Meyer (Hamburg, Germany). All chemicals used were of analytical grade.

### 3.5. Statistical analysis

Results of biochemical analyses are presented as mean value ± standard deviation (S.D.). The differences between control and test groups were analyzed using the Student t-test (significant difference at p ≤ 0.05 confidence level).

## 4. Conclusions

In conclusion, the results of *in vitro* assays of examined laurel extracts expressed significant RSC and protective effects on LP, which was found to be correlated to different compounds, depending on the system of examination. Also, it can be concluded that ethyl acetate proved to be the best solvent for extraction of plant material when *in vitro* antioxidative activity is to be determined. Furthermore, laurel leaf extracts exhibited different activities in relation to the investigated biochemical parameters. On the basis of the results obtained for the values of investigated systems (GSH, GSHPx, LPx, Px, CAT and XOD) after treatment with laurel leaf extracts, as well in combination with CCl_4_, it can be concluded that the investigated extracts showed a certain protective effect, which is more pronounced on the liver than on serum parameters. A possible explanation may be the liver richness in enzymatic systems which can be involved in the pathways of the antioxidant mechanism. The differences observed in the action of particular extracts are probably due to the different contents of flavonoids and some other antioxidant compounds in them. 
